# The Use of Digital and Remote Communication Technologies as a Tool for Multiple Sclerosis Management: Narrative Review

**DOI:** 10.2196/rehab.7805

**Published:** 2018-04-24

**Authors:** Martin Marziniak, Giampaolo Brichetto, Peter Feys, Uta Meyding-Lamadé, Karen Vernon, Sven G Meuth

**Affiliations:** ^1^ Department of Neurology, Isar-Amper-Klinikum Munich-East Haar Germany; ^2^ Scientific Research Area, Italian Multiple Sclerosis Foundation Genova Italy; ^3^ Rehabilitation Research Center, Biomedical Research Center, Faculty of Medicine and Life Sciences, Hasselt University Diepenbeek Belgium; ^4^ Department of Neurology, Krankenhaus Nordwest Frankfurt Germany; ^5^ Department of Neurology, Salford Royal National Health Service Foundation Trust Salford United Kingdom; ^6^ Department of Neurology University of Münster Münster Germany

**Keywords:** communication, eHealth, technology, multiple sclerosis, telehealth, telemedicine, telerehabilitation

## Abstract

Despite recent advances in multiple sclerosis (MS) care, many patients only infrequently access health care services, or are unable to access them easily, for reasons such as mobility restrictions, travel costs, consultation and treatment time constraints, and a lack of locally available MS expert services. Advances in mobile communications have led to the introduction of electronic health (eHealth) technologies, which are helping to improve both access to and the quality of health care services. As the Internet is now readily accessible through smart mobile devices, most people can take advantage of eHealth apps. The development of digital applications and remote communication technologies for patients with MS has increased rapidly in recent years. These apps are intended to complement traditional in-clinic approaches and can bring significant benefits to both patients with MS and health care providers (HCPs). For patients, such eHealth apps have been shown to improve outcomes and increase access to care, disease information, and support. These apps also help patients to participate actively in self-management, for example, by tracking adherence to treatment, changes in bladder and bowel habits, and activity and mood. For HCPs, MS eHealth solutions can simplify the multidisciplinary approaches needed to tailor MS management strategies to individual patients; facilitate remote monitoring of patient symptoms, adverse events, and outcomes; enable the efficient use of limited resources and clinic time; and potentially allow more timely intervention than is possible with scheduled face-to-face visits. These benefits are important because MS is a long-term, multifaceted chronic condition that requires ongoing monitoring, assessment, and management. We identified in the literature 28 eHealth solutions for patients with MS that fall within the four categories of screening and assessment, disease monitoring and self-management, treatment and rehabilitation, and advice and education. We review each solution, focusing on any clinical evidence supporting their use from prospective trials (including ASSESS MS, Deprexis, MSdialog, and the Multiple Sclerosis Performance Test) and consider the opportunities, barriers to adoption, and potential pitfalls of eHealth technologies in routine health care.

## Introduction

Multiple sclerosis (MS) is a chronic disease in which patients’ physical and cognitive abilities often worsen progressively [1]. As well as having to come to terms with these clinical changes, patients frequently find that MS has an impact on social aspects of their lives and those of family members. It is very difficult for a single clinician to manage all areas of the disease; consequently, a multidisciplinary approach is advocated, involving a team of health care professionals (HCPs). To reduce the burden of MS, management strategies must be tailored to individual patients and include multidisciplinary assessment, services, rehabilitation, and appropriate treatment [2].

Important limitations of existing management strategies in chronic diseases such as MS are that clinical evaluation is cross-sectional at particular times, requiring patients to attend regular follow-up visits in MS clinics and comprehensive assessments to be undertaken. Ideally, this should happen at 6- or 12-month intervals, but even at this frequency, mild relapses and disease progression may go unreported. Although more frequent personal consultation could improve disease monitoring, this is probably precluded by time, cost, and geographical constraints. Furthermore, with median survival being as high as 78.6 years in women with MS [3], patients commonly require long-term, multidisciplinary care in both clinical and community settings [1,4]. Despite recent advances in MS care, the availability of expert medical and rehabilitation services may be limited or such services may not be regularly provided owing to a lack of health care reimbursement. Furthermore, many patients cannot access available services because of restricted mobility, fatigue, or travel costs [5-7]. The ability of patients to attend multiple sessions of personalized rehabilitation for specific indications can be constrained by these factors, and long in-patient stays, if necessary, are costly and not widely available. In brief, there are significant implications for patients, their caregivers, and physicians in terms of access to, and provision of, MS health care services [1,4].

**Figure 1:**
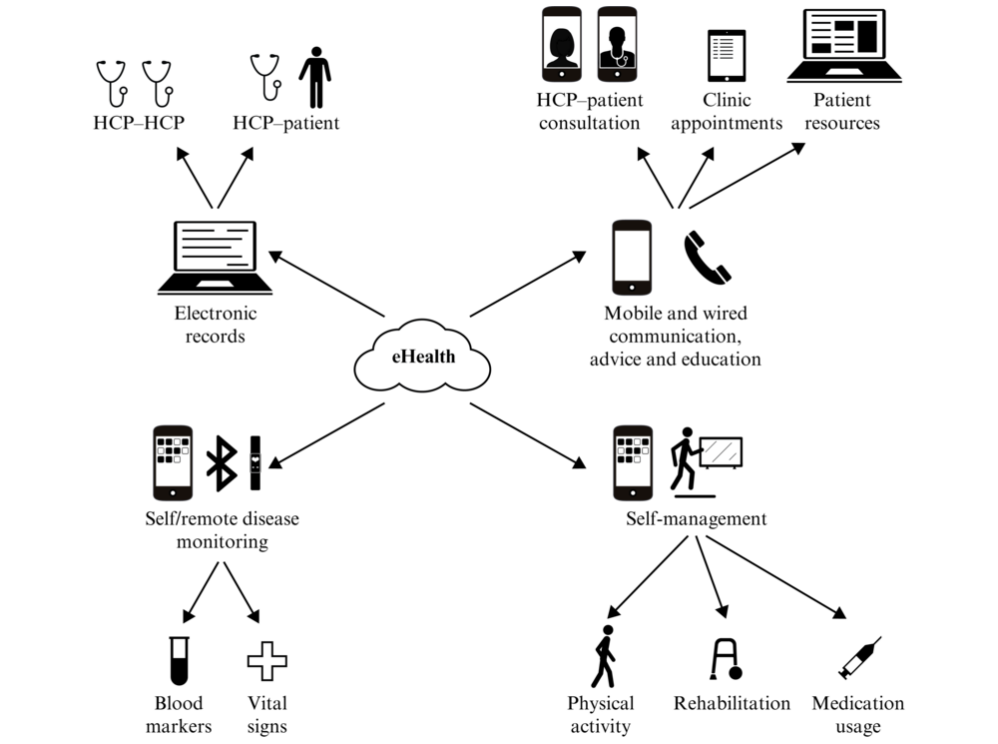
Electronic health (eHealth) technologies and health care. HCP: health care professional.

Electronic health (eHealth) may help to address some of these issues. It has been defined as “an emerging field in the intersection of medical informatics, public health and business, referring to health services and information delivered or enhanced through the Internet and related technologies” [8]. As a subcategory of eHealth, telehealth is of particular note and is defined as “the use of information and communication technologies as a medium for the provision of rehabilitation services to sites or patients who are at a distance from the provider” [1]. eHealth technologies can improve access to health care resources and information by reducing barriers of distance, time, and cost; they can also be deployed to educate and support patients and caregivers in ongoing self-management and to empower patients to become more actively involved in the management and treatment of their disease (Figure 1) [1,9]. Among these, it may be some time before technologies for remote self-monitoring of blood markers in MS are available, but they would be useful. For example, blood tests to monitor disease status in MS form an increasingly important part of disease management, with a requirement for fortnightly monitoring of liver enzyme levels in patients with MS taking teriflunomide. From a service-provider perspective, adoption of such technologies may lead to more efficient use of resources and clinic time, and provide opportunities for monitoring interventions, tracking adverse events, and optimizing therapy that would not be possible with traditional face-to-face approaches alone [1,9].

Many eHealth solutions have been shown to be effective in improving outcomes, in facilitating remote monitoring of symptoms, and in increasing patient engagement, treatment adherence, and access to services and information in chronic diseases such as type 1 diabetes [10] and asthma [11], and in neurological conditions including Parkinson disease [12], suggesting that they may also be effective in MS. Furthermore, by generating prospective, longitudinal, real-world data, eHealth solutions may yield valuable insights into MS disease progression, such as symptoms indicative of relapse. This could also facilitate characterization of different MS disease phenotypes that have been reclassified in recent years to take account of observable active disease [13]. Characterizing a patient’s MS phenotype correctly is crucial, as it impacts directly on decision making regarding treatment.

Opportunities to deploy eHealth have increased significantly in recent years, largely owing to technological advances in mobile communications. For example, at the start of 2017, more than half of the world’s population was using smartphones, nearly two-thirds of the world’s population possessed mobile phones, and more than half of the world’s Web traffic came from mobile phones [14], meaning that many people now have the opportunity to engage with eHealth solutions. A high level of Internet usage among patients with MS has been reported; a survey in 2011 of more than 8500 patients with MS in North America and Canada noted that about 90% of those who responded had access to the Internet or email, and about two-thirds used these at least once daily [15]. Although the situation is unclear among MS physicians specifically, surveys in 2010 and 2013 highlighted a dramatic increase in social media usage from 42% [16] to 75% [17] among practicing physicians in general. Ostensibly, both patients and physicians would seem receptive to the adoption of eHealth solutions.

Although the definition of eHealth can include traditional telephone use, our review will focus on more recent technologies [18] such as the Internet, mobile devices, and apps. The digital and remote technologies developed for individuals with MS that are identified and discussed here pertain to one of four categories: screening and assessment, disease monitoring and self-management, treatment and rehabilitation, and advice and education. There are some overlaps among these four groupings, but they serve to simplify the task of assessing the eHealth landscape and also align broadly with categories used in a recent comprehensive review examining these technologies in a range of clinical conditions [19]. We provide a narrative synthesis of previously published information obtained through a targeted literature search and informed by our personal experience and describe various eHealth solutions, summarizing the clinical evidence supporting their feasibility and use in patients with MS. This review presents a broad perspective on eHealth, a fast-developing field, providing a useful resource to stimulate improved multidisciplinary research projects and services. It will aid HCPs who are interested in integrating eHealth solutions within their clinical practice, offering patients a convenient summary of technologies that have been evaluated clinically, and perhaps helping those developing eHealth solutions in MS to broaden their knowledge of the field. Moreover, the review highlights the need for evaluation of eHealth solutions in MS both in phase 3 clinical trials and in large patient cohorts in real-world settings.

### Screening and Assessment

Few studies have examined the use of digital technologies as MS screening or assessment tools, but they have demonstrated that these technologies provide an efficient alternative to traditional clinic-based methods and are sensitive to capturing important disease information (Table 1). The Multiple Sclerosis Performance Test (MSPT) is a tablet-based app developed to overcome the challenges associated with measuring MS-related disability accurately [20]. The app builds on the assessments in the MS Functional Composite instrument, tracking and providing precise quantitative data on balance, walking speed, visual function, manual dexterity, and cognitive information-processing speed [20]. These outcomes are calculated automatically, and gathering such quantitative performance data provides an opportunity to perform in-depth *post-hoc* analyses of performance patterns. For example, it is possible to gain insights about whether manual dexterity abnormalities are related to grasping, transporting, or releasing [21]. In a prospective clinical validation study that enrolled 51 patients with MS and 49 healthy individuals, data captured with the MSPT app compared favorably with those captured by technicians, and patients reported a high level of acceptance of the tool [20]. The MSPT can be distributed readily to nonexpert clinicians in rural settings and could be adapted for use in patients’ homes, yielding valuable assessment data collected by the patients themselves.

**Table 1:** Digital and remote technologies in multiple sclerosis (MS): screening and assessment. BLCS: Bladder Control Scale; BWCS: Bowel Control Scale; CSI: Cognitive Stability Index; EDSS: Expanded Disability Status Scale; HCP: health care professional; MSPT: Multiple Sclerosis Performance Test; PASAT: Paced Auditory Serial Addition Test; TaDiMuS: Tablet-based Data capture in Multiple Sclerosis.

Tool	Study design	Number of participants	Patient characteristics	Outcomes or applications	Duration of recording	Conclusions
MSPT [20]	Prospective	MSpatients: 51; Healthy controls: 49	Age in years, mean (SD): 46.2 (10.1); EDSS, mean (SD): 3.9 (1.8); Disease duration in years, mean (SD): 12.1 (9.1)	Five performance modules performed by each participant: Walking Speed Test, balance test, Manual Dexterity Test, Processing Speed Test, and Low-Contrast Letter Acuity versus technician testing	Tests repeated twice by each participant during 1 day	MSPT scores were highly reproducible, correlated strongly with technician-administered test scores, discriminated MS from healthy controls and severe from mild MS, and correlated with patient-reported outcomes. Measures of reliability, sensitivity, and clinical meaning for MSPT scores were favorable compared with technician-based testing.
ASSESS MS (Microsoft, Washington, USA; Novartis International AG, Basel, Switzerland) [22]	Prospective	MS patients: 51; Physicians: 12	Age in years, mean (range): 46.0 (23-73); EDSS, mean (range): 3.0 (1.0-7.0); Duration of symptoms in years, mean (range): 14.2 (0.5-47.0)	Classification of motor dysfunction in MS	Tests completed by a HCP within a week (most on a single day)	ASSESS MS is usable and acceptable to both patients and HCPs, generating data of a quality suitable for clinical analysis.
Internet-administered CSI (Headminder Inc, New York, USA)[23,24]	Prospective	MS patients: 40	Age in years, mean (SD): 45 (10.2); Time since diagnosis in years, mean (SD): 10 (7.4)	Measurement of cognitive function over the Internet	PASAT administered 6 times, the score from the last test recorded, then CSI administered. At 14 days, NPsych administered but blinded to PASAT or CSI data.	Compared with NPsych, CSI showed 83% sensitivity and 86% specificity in detecting cognitive impairment, and PASAT showed 28% sensitivity and 86% specificity.
TaDiMuS [25]	Pilot	MS patients: 157	Not reported	Bladder Control Scale BLCS; Bowel Control Scale BWCS	13 months	The mean time taken to complete the BLCS and BWCS was 56.6 s and 39.3 s, respectively. A total of 184 continence test sets (BLCS and BWCS) were completed; an electronic referral for formal continence review was automatically generated 128 times (68.8%) in 108 patients (68.8%), when scores ≥2 in the BLCS or BWCS were achieved.

Another tool, ASSESS MS, which captures depth videos (video images in which each pixel has a three-dimensional, 3D, position) of movement, is under evaluation for the assessment of motor dysfunction in MS within the clinical setting [22]. In a prospective, mixed-methods study that included six neurologists (mean MS experience: 4.3 years) and six nurses (mean MS experience: 2.7 years), ASSESS MS was used to record a predefined set of standard movements in 51 patients [22]. The tool was found to be usable by, and acceptable to, both patients and HCPs, and generated data of sufficient quality for clinical analysis. The tool may also improve accuracy when determining the Expanded Disability Status Scale (EDSS) score [26] by reducing subjectivity, for example, when rating tremor severity during the finger-nose test. Like the MSPT, ASSESS MS is currently at an early stage of development and is used only under the supervision of trained HCPs in a clinical setting, but the potential exists for its adoption in remote eHealth apps.

Although the physician-administered EDSS [26] is the current gold standard for monitoring MS disease severity [27], a patient-administered version could help to capture such data remotely in settings where patients cannot undergo regular physician assessment [27]. With this in mind, a pilot study was conducted [27] that compared an Internet-based version of the telephone-based EDSS [28] with the original physician-administered EDSS [26]. Overall patient satisfaction with the Web-based version was high, and results from it correlated well with those from the physician-administered version [27]. Although further studies are needed to validate the Internet-based tool, these findings suggest it will be invaluable in future long-term monitoring of patients with MS [27].

The heterogeneous nature of MS can make it challenging to measure patients’ cognitive impairment, and it can be time-consuming and expensive to conduct a full battery of neurocognitive tests. Accordingly, an MS-center study enrolled 40 patients with clinically definite MS and subjective cognitive complaints [23] to examine whether cognitive function could be measured remotely using the Internet-administered Cognitive Stability Index (CSI) [24]. The study found the CSI to be as specific as, and more sensitive than, other tools typically used to assess cognitive function in MS, including the neuropsychological battery of tests NPsych and the Paced Auditory Serial Addition Test [23].

Sphincter dysfunction is common in MS [29], but bladder and bowel incontinence can be underreported and poorly managed [25,30]. Furthermore, the use of existing continence-screening tools may be limited in practice by time constraints and physician workload [25]. To address these issues, a cross-platform tool (Tablet-based Data capture in Multiple Sclerosis, TaDiMuS) was developed for use by patients on a tablet computer [25]. In a pilot study, 157 patients completed the TaDiMuS versions of the Bladder Control Scale and the Bowel Control Scale from the MS Quality of Life Inventory [31] while waiting for their appointments (data were captured wirelessly from the waiting room). Scores of ≥2 on either questionnaire generated an automated electronic referral to the clinic’s MS continence nurse [25]. The study confirmed the validity of TaDiMuS as a continence-screening tool, offering physicians an efficient, sensitive, and feasible method of screening patients for bladder and bowel dysfunction [25].

### Disease Monitoring and Self-Management

Many tools have been developed to facilitate disease monitoring and self-management (Table 2). A major challenge facing physicians and patients with MS is how to organize, integrate, and interpret medical data to track disease progression, predict outcomes, and personalize treatment [32]. Accordingly, the University of California, San Francisco, CA, USA developed MS BioScreen, a tablet-based navigation system that integrates data from a patient’s medical records with population-based data to inform physicians about their disease trajectory relative to reference populations and to guide the patient and physician in treatment decisions [32-34].

**Table 2:** Digital and remote technologies in multiple sclerosis (MS): disease monitoring and self-management. EDSS: Expanded Disability Status Scale; MS-HAT: Multiple sclerosis—specific version of Home Automated Telemanagement; MSDS3D: Multiple Sclerosis Documentation System: Three-Dimensional; MSRS-R: Multiple Sclerosis Rating Scale-Revised; N/A: not applicable; RMSS: relapsing-remitting multiple sclerosis; RC: routine care.

Tool	Study design	Number of participants	Patient characteristics	Outcomes or applications	Duration of recording	Conclusions
MS BioScreen (University of California San Francisco MS Centre, San Francisco, USA) [32-34]	N/A	N/A	N/A	Integrate patient information; analyze disease course; facilitate patient engagement	N/A	N/A
MSDS3D [35,36]	N/A	N/A	N/A	Electronic patient-management system that integrates MS registry data	N/A	N/A
MSmonitor (Curavista Health, Geertruidenberg, Netherlands) [37]	Web-based survey	MS patients: 55^a^; RMSS: 38; secondary progressive MS: 11; primary progressive MS: 4; clinically isolated syndrome: 1	Mean age (SD) in years: 46.3 (11.8)	Utilization and meaningfulness of the program’s elements, perceived use of data by neurologists and nurses, and appreciation of care, self-management, and satisfaction	Data collection: January 2013 to April 2013; Survey time: 20 min	In 46% (25/55) of the respondents, the insight into their symptoms and disabilities increased. The overall satisfaction with the program was 3.5 out of 5, and 73% (40/55) of the respondents would recommend the program to other persons with MS.
*move II* (Movisens GmbH, Karlsruhe, Germany) [38]	Pilot	MS patients: 11	Mean (SD) age, in years: 41.0 (9.3). Mean (SD) disease duration, in years: 12.2 (10.7); EDSS 1.0-2.5: n=6; EDSS 3.0-5.0: n=5	Activity parameters	Measurements collected 4 times, each time lasting 10 days and separated by 3 months.	Changes in physical ambulatory activity were captured. *move II* was more responsive to slight disability changes than the clinical measures.
MSRS-R (PatientsLikeMe Inc Cambridge, MA, USA) [39]	Pilot	RRMS patients: 816	Mean (SD) age, in years: 45.9 (9.8); mean (SD) time since diagnosis, in years: 6.6 (6.6)	Measure of MS-related functional disability	2-hour cognitive interview; Web-based survey	The MSRS-R exhibited high internal consistency (Cronbach alpha=.86), correlated highly with existing instruments, (patient-determined disease steps, ρ=.84; Multiple Sclerosis Walking Scale-12, ρ=.83) patient-determined disease stage and relapse burden in the last 2 years. It assesses a number of disability-related domains, while minimizing patient burden.
SymTrac (Novartis International AG, Basel, Switzerland) [40]	N/A	N/A	N/A	Track general well-being and symptoms over time	N/A	N/A
My MS Manager (Multiple Sclerosis Association of America, Cherry Hill, NJ, USA; @Point of Care, Livingston, NJ, USA) [41]	N/A	N/A	N/A	Track disease activity; store medical information; generate charts and reports across various metrics such as treatments, moods, and symptoms	N/A	N/A
MSdialog (Merck Serono, Darmstadt, Germany) [42]	Pilot	MS patients: 42	Mean (SD) age in years: 43.9 (7.6); mean (SD) time since diagnosis, years: 7.0 (6.4); mean (SD) duration of drug treatment, years: 4.8 (4.5)	Health-tracking tool, data from which can be shared with health care providers	6 weeks	82% (32/39) of patients considered MSdialog better than previous methods for tracking their health, and 95% (37/39) would recommend its use.
MS Journal (Tensai Solutions LLC, CA, USA) [43]	N/A	N/A	N/A	Assist with injections	N/A	N/A
myBETAapp (Bayer AG, Leverkusen, Germany) [44]	N/A	N/A	N/A	Schedule, track, and record treatment	N/A	N/A
MS-HAT [45]	Randomized	MS patients: 30; RC: 13; MS-HAT: 17	Mean age (SD) in years—RC: 44.0 (11.8); MS-HAT: 51.0 (9.2). Mean (SD) time since MS onset, in years—RC: 11.9 (9.8); MS-HAT: 18.1 (13.4). Median EDSS (range)—RC: 3.0 (2.0-8.0); MS-HAT: 3.5 (2.0-8.0)	Medication adherence to interferon β-1a	6 months	There were strong correlations between self-reported and objective measures of medication adherence. The majority of patients found the system easy to use, wanted to continue using it after the study ended, and would recommend it to other patients.
MySupport program (Merck Serono, Darmstadt, Germany) [46]	Retrospective	MS patients; MySupport: 604; RC: 2461	Not reported	Persistence with interferon β-1a therapy	24 months	The odds of being on treatment were significantly greater at all time points for patients receiving MySupport versus those receiving routine support only (*P*<.001).

^a^Although 55 patients were surveyed, the sum of patients by multiple sclerosis phenotype is only 54 [30].

The Multiple Sclerosis Documentation System: Three-Dimensional (MSDS3D) was developed following a survey on the inclusion of HCP perspectives on the adoption of eHealth services in neurological practice, which concluded that they were highly appreciated [35]. On the basis of MSDS, the most widely used electronic documentation system in Germany, MSDS3D helps HCPs and patients plan, document, and share clinical data via touchscreen terminals and devices, apps, or a Web browser [35]. Multidimensional data collected by patients and HCPs, including that relating to specific disease-modifying therapies, can be integrated with that from MS registries to provide an innovative resource of long-term follow-up data [36]. In an environment with many disease-modifying therapies, the platform meets a need to monitor clinical outcomes and connect diagnostic and therapeutic processes, thus improving patient care and representing an excellent resource for data mining [35,36].

A separate tool, MSmonitor, is an interactive, Internet-based program for the self-monitoring, self-management, and integrated multidisciplinary care of patients with MS [47]. The original content comprised (1) questionnaires to assess the impact of MS and related quality of life (QoL), fatigue, anxiety, and depression; (2) inventories to capture medication, adherence, and urological symptom data; (3) diaries to record activity, rest periods, micturition, and fluid intake; (4) an *e-consult* functionality to enable patients to contact their physician; and (5) an *e-logbook* [47]. Pilot data suggested that repeated use of MSmonitor led to an increase in health-related QoL and helped patients to self-manage fatigue [47]. In a subsequent survey of 55 patients with MS, MSmonitor has been shown to improve patients’ insights into symptoms and disabilities and improve the quality of nursing care they received [37].

Ambulation is one aspect of physical disability on which the EDSS assessment focuses, particularly at advanced stages of disease. Abnormalities in spatiotemporal parameters that affect walking ability can present in the early stages of MS, and habitual walking performance is sensitive to disease progression and correlates highly with clinical tests of walking capacity and with EDSS score. As such, recording daily activity is considered important for tracking disability progression [48]. A pilot study testing a portable activity-monitoring sensor, designed to gather data on home-based, physical activity (PA) in 11 patients with MS, showed that a simple 3D accelerometer (*move II*) could track fluctuations in daily PA and also tracked disability changes better than EDSS scores [38]. If this finding can be reproduced in a larger group of patients, it may be possible to use the accelerometer to detect signs of worsening disability earlier than when using standard in-clinic measures [38]. Similarly, a study performed in eight patients with MS has demonstrated how data from wireless pressure sensors in patients’ shoes can be combined with Web-based software and mobile technology to detect early signs of deterioration in gait, enabling physicians to intervene rapidly. Data collected were accessible to doctors, patients, and administrators via a Web app [49]. A further study also demonstrated the feasibility of using accelerometers and a multimedia platform to monitor walking function remotely in 25 patients with MS (EDSS score 1.0-6.0) over 2 years and the potential of this approach to capture changes that may indicate deterioration over time [50].

To improve the assessment of functional status in patients with MS, a new patient-reported rating scale, the Multiple Sclerosis Rating Scale-Revised (MSRS-R), was developed, refined, and validated using the Web-based data platform, PatientsLikeMe [39,51]. The MSRS-R was developed to capture disability-related information across a range of domains, rather than focusing on ambulation alone. The MSRS-R exhibits desirable psychometric properties and correlates with existing measures, with the advantage of being more concise than other measures and therefore less burdensome for the patient to complete. Potentially, the MSRS-R, in conjunction with PatientsLikeMe, could provide a valuable source of real-world evidence, encompassing demographic, social networking, treatment, and symptom data [39].

Many apps have been developed to support MS symptom monitoring and disease tracking, but, to date, few have been the subject of published studies. Relapses may not always be tracked because patients forget to report them or because they are not recorded in a patient’s notes. Underreporting of relapses may mean that patients are not receiving the most appropriate treatment, so an app has been developed to address this issue. The Novartis SymTrac app helps to identify when relapses occur by prompting patients to monitor their symptoms and well-being over time, logging information that can be sent automatically to physicians as needed [40,52].

The MS Association of America developed the app My MS Manager for storing medical information, tracking disease activity, generating private reminders, and connecting patients with physicians to share details of their progress [41]. The MSdialog app [53] is an Internet- and mobile-based app designed to capture remote data on clinical and patient-reported outcomes and on self-administration of interferon β-1a [42]. Data from the app are combined with information captured by RebiSmart, an adjustable electronic injection device [53,54]. The app allows patients to create their own health reports and share the information with their physicians. It also tracks trends in treatment adherence and health, and has a reminder function for medication administration and future appointments [53]. In a 6-week pilot study evaluating the app, patients (n=42) found it easy to use and to be superior to their previous health-tracking methods that were mostly handwritten [42].

Nonadherence to MS medications is common and is associated with a number of costs, but monitoring adherence can be challenging, time-consuming, and expensive [45,55]. In addition to the MSdialog app [53], the MSJournal app [43] and myBETAapp [44] have been developed to help patients and caregivers to track injections and injection-site history and to set reminder alerts for injections [43,44]. To test whether telehealth technologies could help to monitor or modify adherence, a study examined adherence to weekly interferon β-1a and daily vitamin D among patients randomized to routine care or to the use of an MS-specific version of the Internet-based Home Automated Telemanagement (HAT) system (MS-HAT) [45]. For 6 months, 30 patients with MS randomized to MS-HAT received text or email reminders to administer injections and a weekly probe asking when they had taken their vitamin D supplements [45]. Although, overall, no major improvements in medication adherence were reported with the MS-HAT system versus routine care, 4 patients (two using MS-HAT and two on routine care) discontinued therapy and did not alert their physicians to their decision: the MS-HAT system detected the discontinuations, allowing timely physician intervention.

**Table 3:** Digital and remote technologies in multiple sclerosis (MS): treatment and rehabilitation. ADL: activities of daily living; BDI: Beck Depression Inventory; EDSS: Expanded Disability Status Scale; GEMS: Guidelines for Exercise in Multiple Sclerosis; HAT: Home Automated Telemanagement; MACFIMS: Minimal Assessment of Cognitive Function in Multiple Sclerosis; MAPSS-MS: Memory, Attention and Problem-Solving Skills for Persons with Multiple Sclerosis; RMSS: relapsing-remitting multiple sclerosis; RC: routine care; tDCS: transcranial direct current stimulation.

Tool	Study design	Number of participants	Patient characteristics	Outcomes or applications	Duration of recording	Conclusions
HAT [56]	Pilot	MS patients: 12	Not reported	Symptom tracking, patient education, exercise regimen instruction and monitoring	12 weeks	Statistically significant improvement in a timed 25-foot walk, 6-min walk, and Berg Balance Scale compared with baseline. Patients were highly satisfied with the service.
MAPSS-MS program [57]	Randomized controlled single-blind	MS patients: 61; MAPSS-MS: 34; Control: 27	Mean (SD) age in years: 47.95 (8.76)	MACFIMS and self-report instruments (use of memory strategies, self-efficacy for control of MS, and neuropsychological competence in ADL) at baseline and after intervention at 2 and 5 months	8 weeks	Both groups improved significantly over time on most measures in the MACFIMS battery, as well as the measures of strategy use and neuropsychological competence in ADL.
Computerized specific training [58]	Randomized controlled double-blind	RRMS patients: 102; attention-specific training: 63; nonspecific training: 39	Mean (SD) age in years: 40.9 (11.5); mean (SD) disease duration in years: 13.0 (8.7); mean (SD) for EDSS: 2.7 (1.5)	Neuropsychological assessment, depression, fatigue, everyday activities, and attentive performance	3 months	A benefit with attention-specific training was observed on the Paced Auditory Serial Addition Test (*P*<.002). However, patient self-reported outcomes did not reveal differences between the training groups.
Home eTraining [59]	Randomized controlled	MS patients: 18; e-training: 9; Hippotherapy: 9	Mean (range) age in years: 45.5 (32-57); mean (range) for EDSS: 3.8 (2-6); mean (range) disease duration in years: 19.0 (1-35)	Static and dynamic balance	12 weeks	Both interventions demonstrated similar and significant improvement in static and dynamic balance capacity.
COGNI-TRAcK (Italian Multiple Sclerosis Foundation, Genoa, Italy) [60]	Pilot	Cognitively impaired MS patients: 16	Mean (SD) age in years: 49.1 (9.1); mean (SD) for EDSS: 3.8 (1.9); mean (SD) disease duration (months): 161.7 (109.6)	Usability, motivation to use, and compliance to treatment	8 weeks	Adherence was 84% (33.4/40). A total of 100% (16/16) of patients felt independent to use the app at home, 75% (12/16) found the exercises interesting, and 81% (13/16) found the exercises useful and were motivated to use the app again.
Web Based Physio [61]	Randomized controlled pilot study	MS patients: 30; Intervention: 15; Control: 25	Mean (SD) age in years: 51.7 (11.2); mean (SD) time since diagnosis (years): 12.7 (9.1); mean (SD) EDSS: 5.9 (0.5)	Mobility, quality of life, and anxiety or depression	12 weeks	No significant between-group difference in primary endpoint (timed 25-foot walk, *P*=.17) or other secondary endpoints except Multiple Sclerosis Impact Scale (*P*=.048). Participants found the website easy to use, convenient, and motivating, and were happy to use it in the future.
MS Invigor8 (University of Southampton, Southampton, UK) [62]	Randomized controlled phase 2 trial	MS patients: 40; MS Invigor8: 23; RC: 17	Mean (SD) age in years—MS Invigor8: 40.1 (17.8); RC: 41.8 (11.4); RRMS (%)—MS Invigor8: 43.5% (10/23); RC: 71% (12/17)	Efficacy in reducing fatigue, feasibility, and cost-effectiveness	10 weeks	There were signiﬁcantly greater improvements in anxiety, depression, and quality-adjusted life-years in patients receiving MS Invigor8.
GEMS [63]	Randomized, controlled pilot study (ongoing)	MS patients: target recruitment: 30	N/A	Efficacy and safety of a home-based, exercise program	4 months	N/A
Deprexis (Gaia AG, Hamburg, Germany) [64]	Randomized, controlled phase 2 trial	MS patients: 90; Deprexis: 45; Waiting list: 45	Mean (SD) age (years)—Deprexis: 45.4 (12.6); Waitlist: 45.2 (10.6); Disability, % patients with walking ability <500 m—Deprexis: 51 (23/45); Waitlist: 49 (22/45). Mean (SD) disease duration in years—Deprexis: 8.2 (7.3); Waitlist: 8.4 (7.6)	BDI	9 weeks	BDI scores decreased in the Deprexis group and increased in the control group (mean difference −4.02 points, 95% CI −7.26 to −0.79; *P*=.02).
Remotely controlled tDCS [65,66]	Pilot	MS patients: 20	Mean (SD) age in years: 51 (9.3); Median (range) EDSS: 4.0 (1.0-8.0)	Feasibility of remote supervision	2 weeks	Across a total of 192 supervised treatment sessions, no session required discontinuation, and no adverse events were reported.

Physicians were pleased to be able to monitor adherence more efficiently than via chart reviews or telephone calls. Furthermore, most patients found the system easy to use, wanted to continue using it after the study, and indicated that they would recommend it to others [45]. Similarly, in the industry-sponsored MySupport program, which provides telephone, text, and website access to patients prescribed interferon β-1a, a retrospective study of anonymized data from 604 patients in the Republic of Ireland found an increased probability of patients using MySupport remaining on treatment compared with a control group of 2461 patients receiving routine care [46].

### Treatment and Rehabilitation

Various treatment and rehabilitation solutions are shown in Table 3. The Internet-based HAT system was designed for use in patients’ homes to monitor symptoms and educate them about their condition [56]. It was also developed to provide step-by-step instructions on how to follow a tailored exercise regimen, to monitor exercise compliance, and to adapt the exercise regimen based on performance [56]. A 12-week pilot study that enrolled 12 patients with MS provided a preliminary demonstration of the feasibility of the HAT system and its potential for use on other devices such as tablets and mobile phones. Its ease of use and convenience were considered particularly beneficial for patients who may be reluctant or unable to visit a physician frequently [56].

The use of other home-based technologies has been explored in a variety of MS rehabilitation settings, including Internet- or computer-assisted training to enhance cognitive performance [57,67], attention [58], and balance, posture, and strength [59]. The Memory, Attention, and Problem Solving Skills for Persons with MS (MAPSS-MS) program is a computer-assisted cognitive rehabilitation intervention for enhancing cognitive function in patients with MS [57]. In the 8-week, single-blind, randomized controlled MAPSS-MS study involving 61 patients with MS, significant improvements in cognition were observed with the MAPSS-MS program, and it was found to be feasible to use and well accepted by patients [57]. COGNI-TRAcK, a self-administered cognitive training app, has also been shown to provide intensive and personalized cognitive rehabilitation [60,68]. In 16 patients with MS and cognitive impairment who underwent an 8-week intervention at home, COGNI-TRAcK was found to be highly usable, motivating, and well accepted by users [60]. COGNI-TRAcK was also evaluated in 28 patients with MS and cognitive impairment to determine the effects of adaptive versus nonadaptive cognitive training. Adaptive training involved increasing or decreasing the difficulty level of an exercise based on whether a participant completed preceding exercises correctly. COGNI-TRAcK was shown to be suitable for administering personalized training to patients with cognitive impairment. It also demonstrated that an adaptive work load is crucial for determining the effectiveness of cognitive treatment, with only patients in the adaptive group showing improvements in cognitive function on study and at 6-month follow-up [68].

A double-blind, randomized controlled study has evaluated a home-based computerized program for retraining attention dysfunction under the supervision of a caregiver in 102 patients with relapsing-remitting MS [58]. Compared with nonspecific training, the attention-dysfunction-specific training resulted in some improvements exclusively in tasks of sustained attention, although patient-reported outcomes did not reveal differences between the groups [58]. Balance was also shown to be improved using the Internet-based program Home eTraining. In a randomized controlled study that enrolled 18 patients with MS, eTraining demonstrated significant improvements in static and dynamic balance that were similar to those resulting from hippotherapy [59].

Home-based technologies have also been used for Web-based physiotherapy exercises [61,69], physical telerehabilitation [70], and physical-activity-targeted behavioral interventions [71,72]. Web Based Physio, developed by the University of Glasgow, Glasgow, United Kingdom, provides Web-based physiotherapy exercise programs personalized for individual patients with long-term conditions including MS, with the aim of allowing patients to self-manage their condition [69]. The effectiveness of this individualized, Internet-based physiotherapy program was evaluated in a 12-week randomized controlled pilot study in community-dwelling adults moderately affected by MS (EDSS score 5.0-6.5). Although there was no significant difference in the primary outcome measure (timed 25-foot walk), patients found the website easy to use, convenient, and motivating, and indicated a willingness to use it in the future. A fully powered, definitive randomized controlled trial is planned to determine the tool’s effectiveness [61].

Another 12-week study assessed feasibility of use, patient acceptance, and magnitude of clinical benefit of home-based physical telerehabilitation in 12 individuals with MS (75%, 9/12, had self-reported moderate MS) who received a tailored rehabilitative exercise program [70]. Home-based physical telerehabilitation was shown to significantly improve functional outcomes including walking and balance. Internet-delivered behavioral interventions have also demonstrated an increase in activity among patients with MS. A 3-month randomized controlled study evaluating an Internet-delivered and theory-based behavioral intervention that was supplemented with video coaching in 45 patients with MS showed a large increase in PA after 12 weeks that was sustained over 3 months [72].

Computer- or gaming-based systems, such as the Nintendo Wii Fit console or Kinect motion sensor, may offer the potential for telerehabilitation applications in patients with MS because patients enjoy these exercises and find them motivating [73]. Although some of these applications have demonstrated significant beneficial effects, the results of others have been mixed. For example, the Nintendo Wii platform appears to stimulate the postural control system only in the frontal plane and not the sagittal plane [74]. Thus, although available games are beneficial in some settings, they will be more effective if tailored to the type and severity of impairments present in individual patients with MS and adapted to offer Internet-assisted monitoring [74,75]. In general, the success of Nintendo Wii or *exergaming* technologies in randomized controlled clinical studies has also been mixed [76-78], but in a 24-week diffusion tensor imaging study, modifications in the microstructure of superior cerebellar peduncles were observed following 12 weeks of Nintendo Wii balance-board training [79]. These changes correlated with clinical improvements in participants’ balance, suggesting that high-intensity, task-oriented exercises could induce favorable, myelin-related microstructural changes in the brains of patients with MS [79].

To address the potential issue of patients performing their rehabilitation exercises incorrectly, a comprehensive system is in development that combines weekly face-to-face clinic sessions with remotely supervised exercise training at home, using a Web-based platform and tracking tool that analyzes and corrects patients’ positions in real time [80]. This tool is currently being validated, and preliminary results indicate that the system can be used effectively by patients and HCPs [80].

In light of the success of telephone-administered interventions for improving various MS-associated symptoms and QoL [81-85], several studies have examined (or will examine) whether interactive telehealth interventions can improve MS-associated anxiety, cognitive function, mood, fatigue, impact, pain, QoL, and sleep quality [62-64,86-90]. These include a Web-based self-help program (Deprexis) that combines cognitive behavioral therapy (CBT) with mobile platform and dialog technology and has proven efficacy in treating depression [64,91]; an Internet-based CBT program (MS Invigor8) administered with or without email support to help reduce fatigue symptoms [62,90]; MS-specific multimedia software that delivers a meditation course designed to decrease anxiety, depression, and fatigue, as well as improve quality of sleep and QoL [88]; the project Guidelines for Exercise in Multiple Sclerosis, an interactive, guidelines-based exercise program aimed at improving MS symptoms and QoL [63]; and physical exercise e-training programs that demonstrate positive and significant effects on muscle strength, lung function, and sports activity, but not on QoL or fatigue [92,93]. Perhaps the most robust of these studies was a 9-week randomized trial conducted in patients with MS who had self-reported depression symptoms (N=90) [64]. Patients received the intervention (Deprexis [91]) or remained on a waiting list (control) for 9 weeks, and over the course of the study, use of Deprexis significantly reduced Beck Depression Inventory scores, whereas scores increased in the control group. These results highlight the utility of Web-based intervention programs, especially for patients who cannot attend or do not like to participate in treatment sessions regularly [64].

Home-based, but clinician-supervised, technologies have also been examined, such as the remotely supervised *self- or proxy-administration protocol* for home delivery of transcranial direct current stimulation (tDCS) [65]. Across 192 supervised treatment sessions, remotely supervised protocol adherence was greater than that observed in clinic-based delivery studies. Furthermore, there were no reported discontinuations or adverse effects. Thus, remotely supervised tDCS could be used to expand patient access to this potential treatment option [65].

### Advice and Education

Several apps have been developed to provide advice and education to individuals living with MS, including the MS Buddy app [94] and the MS self app, which, in addition to providing MS-related information, can synchronize with Fitbit devices (Table 4) [95].

**Table 4:** Digital and remote technologies in multiple sclerosis (MS): advice, support, and education. MCCO: Mellen Center Care Online; N/A: not applicable.

Tool	Study design	Number of participants	Patient characteristics	Outcomes or applications	Duration of recording	Conclusions
MS Buddy (Healthline Networks Inc., San Francisco, USA) [94]	N/A	N/A	N/A	An app for discovering support and getting advice from an MS peer	N/A	N/A
MS self (Acorda Therapeutics Inc., New York, USA) [95]	N/A	N/A	N/A	An app designed to help patients manage their MS	N/A	N/A
My MS Manager (Multiple Sclerosis Association of America, Cherry Hill, NJ, USA; @Point of Care, Livingston, NJ, USA) [41]	N/A	N/A	N/A	Provides advice and support	N/A	N/A
Deprexis (Gaia AG, Hamburg, Germany) [64]	Randomized, controlled phase 2 trial	MS patients: 90; Deprexis: 45; Waiting list: 45	Mean (SD) age, in years—Deprexis: 45.4 (12.6); Waitlist: 45.2 (10.6). Disability, % patients with walking ability <500 m—Deprexis: 51; Waitlist: 49. Mean (SD) disease duration, in years—Deprexis: 8.2 (7.3); Waitlist: 8.4 (7.6)	Web-based psychoeducation; Beck Depression Inventory	9 weeks	N/A
MCCO system (Cleveland Clinic, Cleveland, OH, USA) [97]	Randomized controlled	MS patients: 206; MCCO-original: 104; MCCO-enhanced: 102	Mean age (SD), years—MCCO-original: 48.1 (9.7); MCCO-enhanced: 48.1 (9.1) Mean Incapacity Status Scale (SD)—MCCO-original: 12.3 (9.2); MCCO-enhanced: 12.7 (8.2)	Compare MCCO-original versus MCCO-enhanced	12 months	No differences in patient- or physician-reported outcomes were reported.

The Mellen Center Care Online (MCCO) secure Internet-based portal was developed in 1998 to empower patients to participate more actively in their own health care [96,97]. It is designed to help address patients’ concerns, enhance communication between patients and physicians, provide links to patient information about MS symptoms, and allow patients to monitor changes in their disease status and prepare for upcoming health care visits [96,97]. The system functionality was later expanded to include a self-monitoring and self-management component that allowed patients to assess their MS symptoms and receive graphical feedback and evaluate symptom changes to make decisions about how to respond to them [97]. Apps that have been developed for disease monitoring or rehabilitation also provide patient advice and support, including the My MS Manager app [41] and Deprexis [91]. In addition, patients with life-changing illnesses (including MS) who use Web-based, quantitative, personal research platforms such as PatientsLikeMe report important benefits such as being able to learn more about their symptoms and understand potential side effects of their treatment [51].

## Discussion

### Summary

**Figure 2:**
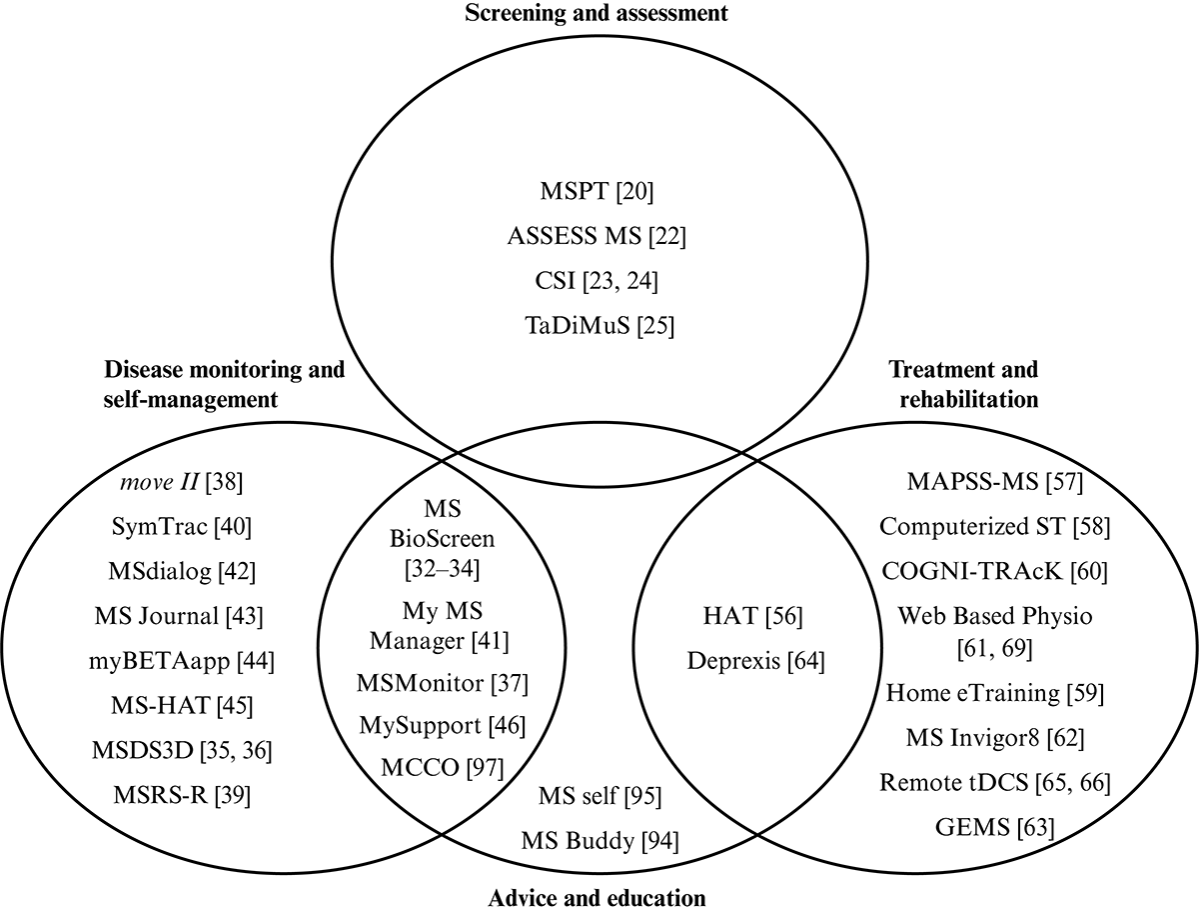
Overview of electronic health (eHealth) technologies applied in multiple sclerosis. CSI: Cognitive Stability Index; GEMS: Guidelines for Exercise in Multiple Sclerosis; HAT: Home Automated Telemanagement; MCCO: Mellen Center Care Online; MS: multiple sclerosis; MS-HAT: Multiple Sclerosis—specific version of Home Automated Telemanagement; MSDS3D: Multiple Sclerosis Documentation System: Three-Dimensional; MSPT: Multiple Sclerosis Performance Test; MSRS-R, Multiple Sclerosis Rating Scale-Revised; TaDiMuS: Tablet-based Data capture in Multiple Sclerosis; MAPSS-MS: Memory, Attention and Problem-Solving Skills for Persons with Multiple Sclerosis; ST: specific training; and tDCS: transcranial direct current stimulation.

There has been a rapid increase in the development, testing, and use of digital and remote communication technologies in MS in recent years, with numerous studies demonstrating the value of these tools. The MS eHealth solutions identified here (Figure 2) mostly support disease monitoring, self-management, treatment, and rehabilitation. A few of these also offer patient advice and education, although apps have also been developed specifically for this. Fewer technologies address screening and remote assessment, and it may be that this area has the greatest scope for the development of new tools in the future.

### Principal Findings

Of the 28 eHealth solutions discussed here, 14 are Web-based (Computerized Specific Training, CSI, Deprexis, HAT, Home eTraining, MAPSS-MS, MCCO, MSDS3D, MS-HAT, MSInvigor8, MSMonitor, MSRS-R, MySupport, and Web Based Physio), and 11 are apps (COGNI-TRAcK, MS Bioscreen, MS Buddy, MSdialog, MS Journal, MSPT, MS self, MyBETAapp, My MS Manager, SymTrac, and TaDiMuS). The remaining three use home-based technologies found in games consoles (ASSESS MS and *move II*) or specialist equipment (Remote tDCS). Apps are more represented than other platforms among solutions that relate to disease monitoring and self-management, and Web-based solutions account for more of the treatment and rehabilitation solutions than do apps. This trend probably reflects the frequency of use (and hence portability) and data burden (and therefore bandwidth) associated with different solutions.

Although the development of digital and remote communication technologies is welcome, their true value can be realized only if patients and physicians jointly engage with them. Despite the solid evidence base demonstrating the success of telephone-based interventions, many patients with MS do not receive this relatively basic therapy. Thus, it may be helpful to understand what barriers impede delivery of telephone-based rehabilitation before attempts are made to roll out more technologically advanced telehealth solutions on a large scale. It is likely that factors such as mobile Internet access, available bandwidth in remote geographical regions, and cost are all barriers to global uptake of eHealth solutions and that other factors such as availability of specialist clinicians and adaptation of established solutions to suit local situations (language and cultural and educational issues) will need to be overcome. Irrespective of the setting, it is likely that educational programs will be needed as part of training within health care systems to help clinicians understand the value that various communication technologies could bring to routine patient assessment and to ensure that any technologies that are adopted are applied with standardized methods and reporting.

Encouragingly, studies in patients with MS that have examined the use and acceptance of communication technologies suggest that adoption is unlikely to be a major issue; proportionally more patients with MS than in the general population use the Internet in the United States [98], and the majority of German patients at MS specialist centers regularly use modern communication technologies and are happy to use them to communicate with their physicians and other HCPs, including MS nurses [99]. Notably, most patients participating in the North American Research Committee on Multiple Sclerosis Registry (2011) reported that the Internet was their first source for health information [15], and studies examining the browsing habits of patients with MS show that the most-viewed topics related to *understanding the disease* and *treatments* [100]. Finally, patients express high levels of satisfaction with home telehealth monitoring [101] and have high levels of acceptance of systems such as MS-HAT [102], finding them easy to use [45].

There are aspects of these technologies with which patients are uncomfortable. Some patients were intimidated by the Nintendo Wii Fit owing to concerns about falling, and some disliked exergaming feedback because it reminded them of their impairments [103]. Furthermore, some were unsure how to use a video game [73], so appropriate training is needed. Particular challenges for those with MS using Web-based technologies include difficulties in reading website text, problems with flashing or moving objects, and operation of a mouse or keyboard because of dexterity issues [96]. Patients can also be unaware of accessibility features that facilitate navigation of websites [96]. Finally, patients may dislike interventions that are overly intrusive, and they may have concerns about security issues associated with remote transmission of personal data. This particular issue may be resolved with emerging security technologies such as Integrated Circuit Metric [104], but these will almost certainly need to be safeguarded with appropriate legislation.

Digital technologies should complement but not replace face-to-face consultations and should thus be welcomed by physicians, especially if they reduce the burden on health care services. It is possible that the high volume of data that certain tools may generate could discourage their adoption clinically, and it is to be determined whether data gathered remotely provide a better picture of disease status than standard follow-up visits and whether these technologies are associated with improvements in long-term patient outcomes. Consensus about which technologies are most useful and cost-effective is lacking, and physicians may be reluctant to invest the time needed to become familiar with such tools, irrespective of any potential efficiency they offer.

In the future, digital and remote technologies may expand to other uses; for example, Web-based platforms such as PatientsLikeMe have been used to develop disease-specific instruments (ie, the MSRS-R) [39], and the Internet-based Dutch Multiple Sclerosis Study used the Internet to recruit patients, monitor symptoms, and capture long-term disease progression data in real-world settings [105]. Similarly, interactive technologies such as the Web-based patient-management system MSDS3D may become increasingly common [106]. There are ongoing initiatives to develop transparent systems for disease monitoring and self-management in MS, such as Remote Assessment of Disease and Relapse in Central Nervous Disorders. This international research project is applying wearable devices and mobile phone technology to develop ways of measuring major depressive disorders, epilepsy, and MS [107]. There is also the MAPPING-MS initiative, a mobile health intervention that will deliver self-management strategies in patients with MS [108]. It seems likely that elements of the many different apps, Web-based tools, and remote monitoring systems that have already been adopted or are in development will become part of larger integrated systems that facilitate eHealth care conveniently for both patients and HCPs.

### Conclusions

In conclusion, many digital and remote communication technology applications have been developed for patients with MS, and evidence is accumulating for the benefits some of these can bring compared with, and complementary to, traditional in-clinic approaches. Most tools focus on disease monitoring, self-management, treatment, and rehabilitation, so greater emphasis could be placed on developing tools dedicated to screening and assessment. However, irrespective of the eHealth solution under consideration, data from large, controlled, multicenter trials are lacking (only MSInvigor8 and Deprexis were phase 2 trials), so it is difficult to draw objective conclusions about clinical benefits associated with each technology. Evaluation of eHealth solutions in phase 3 trials may be precluded by cost, in which case prospective surveys in real-world settings [39] or large, retrospective database analyses [46] may be the most pragmatic means of evaluation. Ultimately, the long-term benefits afforded to patients and clinicians by any of these technologies will need to be established before their widespread adoption is likely.

## Abbreviations

ADL: activities of daily living
BDI: Beck Depression Inventory
BLCS: Bladder Control Scale
BWCS: Bowel Control Scale
CBT: cognitive behavioral therapy
CSI: Cognitive Stability Index
EDSS: Expanded Disability Status Scale
eHealth: electronic health
GEMS: Guidelines for Exercise in Multiple Sclerosis
HAT: Home Automated Telemanagement
HCP: health care professional
MACFIMS: Minimal Assessment of Cognitive Function in Multiple Sclerosis
MAPSS-MS: Memory, Attention and Problem-Solving Skills for Persons with Multiple Sclerosis
MCCO: Mellen Center Care Online
MS: multiple sclerosis
MSDS3D: Multiple Sclerosis Documentation System: Three-Dimensional
MS-HAT: Multiple Sclerosis—specific version of Home Automated Telemanagement
MSPT: Multiple Sclerosis Performance Test
MSRS-R: Multiple Sclerosis Rating Scale-Revised
PA: physical activity
PASAT: Paced Auditory Serial Addition Test
QoL: quality of life
RC: routine care
RRMS: relapsing-remitting multiple sclerosis
TaDiMuS: Tablet-based Data capture in Multiple Sclerosis
tDCS: transcranial direct current stimulation
3D: three-dimensional
